# The nomogram of contrast-enhanced ultrasound-targeted fusion biopsy predicts the pathology upgrade in prostate cancer

**DOI:** 10.3389/fonc.2025.1632501

**Published:** 2025-11-17

**Authors:** Jianhui Cao, Bin Feng, Junbiao Zheng

**Affiliations:** 1 Department of Ultrasound, The First People’s Hospital of Jiashan, Zhejiang, China; 2 Department of Urology, The First People’s Hospital of Jiashan, Zhejiang, China

**Keywords:** prostate cancer, contrast enhanced ultrasound, biopsy, Gleason grade group, Gleason grade group upgrading

## Abstract

**Aim:**

The aim of this study was to analyze the consistency between the pathology of combined systematic and contrast-enhanced ultrasound (CEUS)-targeted prostate biopsy and radical prostatectomy (RP) by building a nomogram to predict the pathology upgrade of Gleason grade group (GGG).

**Methods:**

A total of 113 participants with prostate cancer with combined systematic and CEUS-targeted prostate biopsy followed by RP were recruited between January 2021 and November 2024. The Kappa coefficient of pre- and post-RP GGG was calculated. The independent predictors for pathology upgrade were screened using logistic regression and then applied to build a nomogram for pathology upgrade prediction. The performance of the nomogram was assessed by receiver operating characteristic analysis, calibration curve analysis, and decision curve analysis.

**Result:**

Among 113 participants, 25 (22.1%) Gleason grade group upgrading (GGGU) and 88 (77.9%) non-GGGU cases were found. Moderate consistency of prostate cancer GGG between combined systematic and CEUS-targeted prostate biopsy and final RP pathology was found (Kappa = 0.46, *p <* 0.01). The primary biopsy Gleason Score [odds ratio (OR) = 0.22, p < 0.01] and the greatest percentage of cancer in a single core (OR = 1.04, *p <* 0.01) were independent predictors for post-RP GGGU. The area under the curve of the established nomogram reached 0.83, and the calibration curve showed a robust result.

**Conclusion:**

The nomogram integrating CEUS-targeted fusion biopsy variables effectively predicted the risk of pathology upgrade in prostate cancer, which showed potential to guide clinicians in optimizing surgical management in patients with prostate cancer.

## Background

Prostate cancer has become the most common malignancy in elderly men, with an increased incidence worldwide (1). Risk stratification and treatment strategies for prostate cancer are determined based on patients’ clinical characteristics, such as age, prostate-specific antigen (PSA), imaging findings such as magnetic resonance imaging (MRI), and preoperative prostate biopsy Gleason grade group (GGG) (2). Since prostate cancer is multifocal and heterogeneous, systematic biopsy is the most commonly used method for a clinical preoperative diagnosis of prostate cancer (3). However, there were cases where the GGG of prostate cancer through biopsy appeared to be upgraded after radical prostatectomy (RP), leading to inadequate treatment. Therefore, targeted prostate biopsy guided by multiple imaging modalities such as MRI and ultrasound has been proposed to improve the diagnostic accuracy rate of prostate cancer before RP (4). MRI-guided targeted biopsy is currently recognized as the reference standard for preoperative grading of prostate cancer (5). However, relevant studies have shown that the Gleason grade group upgrading (GGGU) rate of patients with prostate cancer by MRI-guided biopsy undergoing RP was still high (31.6%–35.0%) (6, 7). Meanwhile, MRI-guided biopsy is expensive and time-consuming and requires a puncture technique and surgical equipment, making it difficult to apply in primary hospitals.

Ultrasound has been widely applied in diagnosis and treatment, especially contrast-enhanced ultrasound (CEUS), which includes microbubbles to improve the sensitivity of tumor microvascular detection (8). CEUS-targeted biopsy is convenient and uses real-time dynamic imaging, while targeted puncture may find more prostate cancer foci than the systematic puncture method (9). Relevant studies have shown that prostate cancer tissues detected by CEUS-targeted fusion biopsy have higher Gleason Score, thus improving the detection rate of prostate cancer (10, 11). Therefore, combined systematic and CEUS-targeted prostate biopsy can improve the accuracy of preoperative assessment of prostate cancer.

This study applied the GGG scale according to the International Society of Urological Pathology (ISUP) 2014 edition (12), and 113 patients with prostate cancer with combined systematic and CEUS-targeted prostate biopsy followed by RP were retrospectively enrolled. The consistency of GGG before and after RP was calculated, and a nomogram for predicting GGGU after RP was established and internally validated, in order to enable clinicians to make accurate decisions for patients with different grades of prostate cancers.

## Materials and methods

### Study population

A total of 113 patients with prostate cancer who underwent combined systematic and CEUS-targeted prostate biopsy followed by RP at the First People’s Hospital of Jiashan, Zhejiang Province, China, were randomly enrolled from January 2021 to November 2024. Inclusion criteria were as follows: (1) patients had no obvious contraindications to puncture or surgery and had no history of prostate biopsy; (2) the puncture protocol was combined systematic and CEUS-targeted prostate biopsy; (3) the pathological diagnosis of prostate follicular adenocarcinoma was obtained; and (4) patients underwent RP within 90 days of prostate biopsy. Exclusion criteria were as follows: (1) imaging examinations suggesting bone metastasis and/or metastasis to other organs; (2) patients allergic to ultrasound contrast agents; (3) patients have acute infectious or febrile symptoms with severe cardiopulmonary insufficiency or bleeding tendency; and (4) missing or incomplete data. This study was approved by the Ethics Committee of the First People’s Hospital of Jiashan (Ethics No. KT2023001). Informed consent was obtained from all patients.

### Instruments and protocols

**Figure 1:**
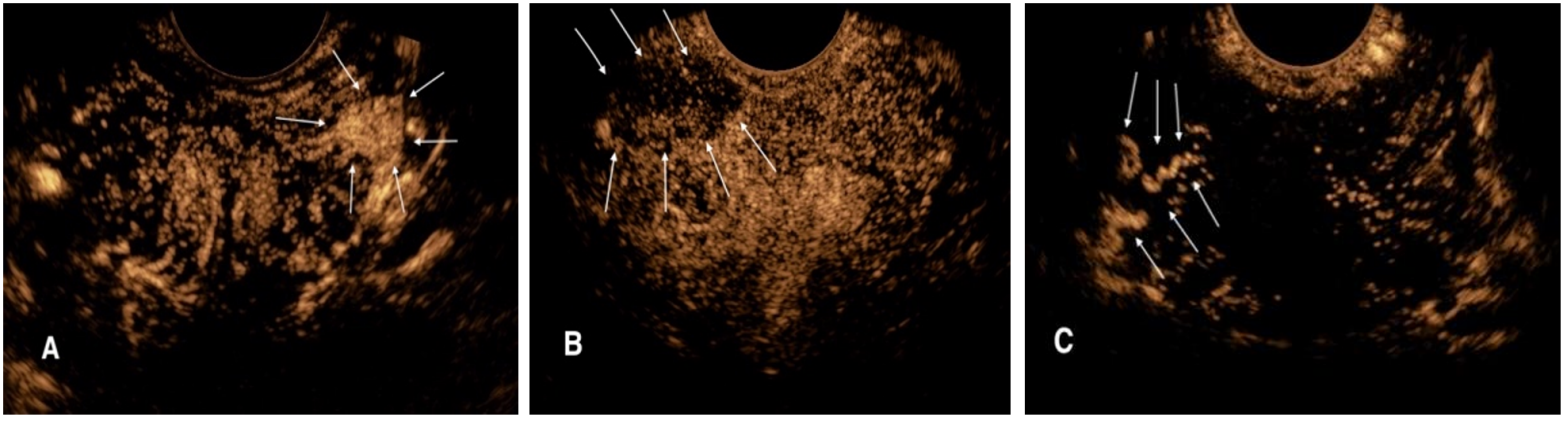
CEUS diagnostic criteria for suspicious prostate lesions. **(A)** Rapid high enhancement lesion in the left peripheral arterial phase of prostate (arrow). **(B)** Low enhancement lesion with unclear boundary in the right peripheral arterial phase of prostate (arrow). **(C)** Abnormal vascular enhancement in the right peripheral arterial stage of prostate (arrow).

Ultrasonography examinations were performed using the Logiq E9 (GE Medical, Wauwatosa, WI) ultrasound system with an IC5–9 endorectal probe at a frequency of 4–9 MHz. After routine ultrasound was completed, CEUS mode was activated and performed at three levels of the prostate: base, midgland, and apex, and if a hypoechoic nodule was found in the prostate during routine ultrasound, CEUS would be performed at that section to monitor the perfusion of the nodule. Sulfur hexafluoride (SonoVue; Bracco, Italy) was used as the contrast agent. SonoVue suspension (25 mg, lyophilized powder) and saline solution (5 mL, 0.9% sodium chloride) were prepared and shaken consistently. During the examination, 2.4 mL of contrast medium was injected intravenously and then flushed with 5 mL of saline. The injection was started synchronously with the timing from 0 second, and a 90-second-record was stored. The CEUS diagnostic criteria for suspicious prostate lesions were as follows: (1) localized rapid hyper-enhancement in the arterial phase; (2) low-enhancement lesions with unclear boundaries; and (3) abnormal vascular enhancement (**Figure 1**).

Prostate puncture biopsy was performed using the Mylab 90 (Esaote SpA, Genoa, Italy) scanner with a TRT33 biplane probe. The transperineal biopsy approach was chosen and a standard 10-core systematic puncture procedure was performed. For patients with suspicious lesions on CEUS, one to three needle samples were obtained for target lesions in **Figure 1**. If the CEUS suspicious prostate lesions were coincident with the systematic puncture point, then this area will not be repeated. All prostate puncture biopsies and CEUS were performed by a sonographer with more than 10 years’ experience in prostate ultrasonography and biopsy; the sonographer was blinded to the patients’ clinical information.

The pathological diagnosis was based on the 2014 ISUP diagnostic criteria for Gleason scoring and grading system for prostatic follicular adenocarcinoma (12). The grading system was defined as follows: GGG1 [Gleason score (GS) ≤ 6], GGG2 (GS 3 + 4 = 7), GGG3 (GS 4 + 3 = 7), GGG4 (GS = 8: 4 + 4, 3 + 5, or 5 + 3), and GGG5 (GS = 9 or 10). GGGU was defined as the higher post-RP pathological GGG than biopsy GGG. All prostate biopsy specimens and RP specimens were diagnosed by a pathologist with at least 10 years’ experience in prostate pathology, who was blinded to the patients’ clinical information.

### Statistical analysis

All data analysis was performed using SPSS 22.0 and R 3.6.0. Categorical variables were expressed as frequency and percentage (%), while continuous variables were expressed as average ± standard deviation or median (M) and interquartile range (IQR); Kruskal–Wallis test was used for non-parametric tests. The Kappa test was employed to evaluate the concordance between the pathological GGG of biopsy and that of RP specimen. Subsequently, univariate logistic regression was conducted on relevant clinicopathological indexes to identify the independent influencing factors for GGGU of RP, and a prediction model of nomogram was constructed. Firth’s bias-reduced logistic regression was used to mitigate small-sample bias. The predictive efficacy of the model was evaluated using the receiver operating characteristic (ROC) curve with the area under the curve (AUC), calibration curves were plotted to assess its consistency, and internal validation was assessed by bootstrap. Sensitivity analysis of leave-one-out cross-validation was performed to check the model’s robustness. Statistical significance was determined at the *p <* 0.05 level.

## Results

### Comparison of characteristics between different GGG groups

This study enrolled 113 RP cases, with 25 GGGU cases after RP (22.1%, average age 73.8 ± 4.4 years), 24 GGG degrading cases (21.2%, average age 73.9 ± 5.3 years), and 64 cases remaining the same (56.7%, average age 72.2 ± 5.8 years). These three cohorts had comparable baseline age, total PSA (tPSA), PSA density [PSAD = tPSA/prostate volume, while prostate volume was calculated as follows: prostate’s right–left diameter (cm) × vertical diameter (cm) × anteroposterior diameter (cm) × 0.52], secondary biopsy Gleason score (sGS), and number of positive cores, while the primary biopsy Gleason score (pGS) and the greatest percentage of cancer in a single core (GPC, assessed by a pathologist with at least 10 years’ experience in prostate pathology through microscopic vision) were significantly different between groups (*p* = 0.01 and *p <* 0.01, respectively) (**Table 1**).

**Table 1:** Basic characteristics of 113 patients with prostate cancer included in the study.

Baseline variables	Upgrading (n = 25)	Concordance (n = 64)	Degrading (n = 24)	Total (n = 113)	*p*-value
Age (years)	73.8 ± 4.4	72.2 ± 5.8	73.9 ± 5.3	72.9 ± 5.5	0.28
tPSA, M (IQR) (ng/mL)	12.3 (8.9, 26.7)	11.7 (7.3, 20.0)	13.4 (11.5, 20.1)	12.6 (7.8, 21.1)	0.29
PSAD, M (IQR) (ng/mL^2^)	0.5 (0.3, 0.9)	0.4 (0.2, 0.7)	0.5 (0.3, 0.9)	0.4 (0.3, 0.8)	0.31
pGS	3.4 ± 0.5	3.7 ± 0.7	3.4 ± 0.5	3.6 ± 0.7	0.01
sGS	3.5 ± 0.5	3.7 ± 0.8	3.8 ± 0.6	3.7 ± 0.7	0.26
GPC (%)	61.4 ± 21.8	42.2 ± 25.8	42.7 ± 24.8	46.5 ± 25.8	<0.01
Number of positive cores	5.0 ± 2.4	3.8 ± 2.2	4.4 ± 2.2	4.2 ± 2.3	0.06

### The consistency between pathology finding of combined systematic and CEUS-targeted prostate biopsy and RP

The GGG of prostate biopsy was set as baseline and compared to the GGG of RP (**Table 2**). The Kappa test demonstrated that GGG between prostate biopsy and RP is of good consistency (Kappa = 0.46, *p* < 0.01).

**Table 2:** GGG distribution of 113 patients.

Prostate biopsy	RP					
	1	2	3	4	5	Total
1	23	6	2	1	1	33
2	4	8	5	1	0	18
3	1	5	8	1	2	17
4	1	3	7	8	6	25
5	0	0	1	2	17	20
Total	29	22	23	13	26	113

**Figure 2:**
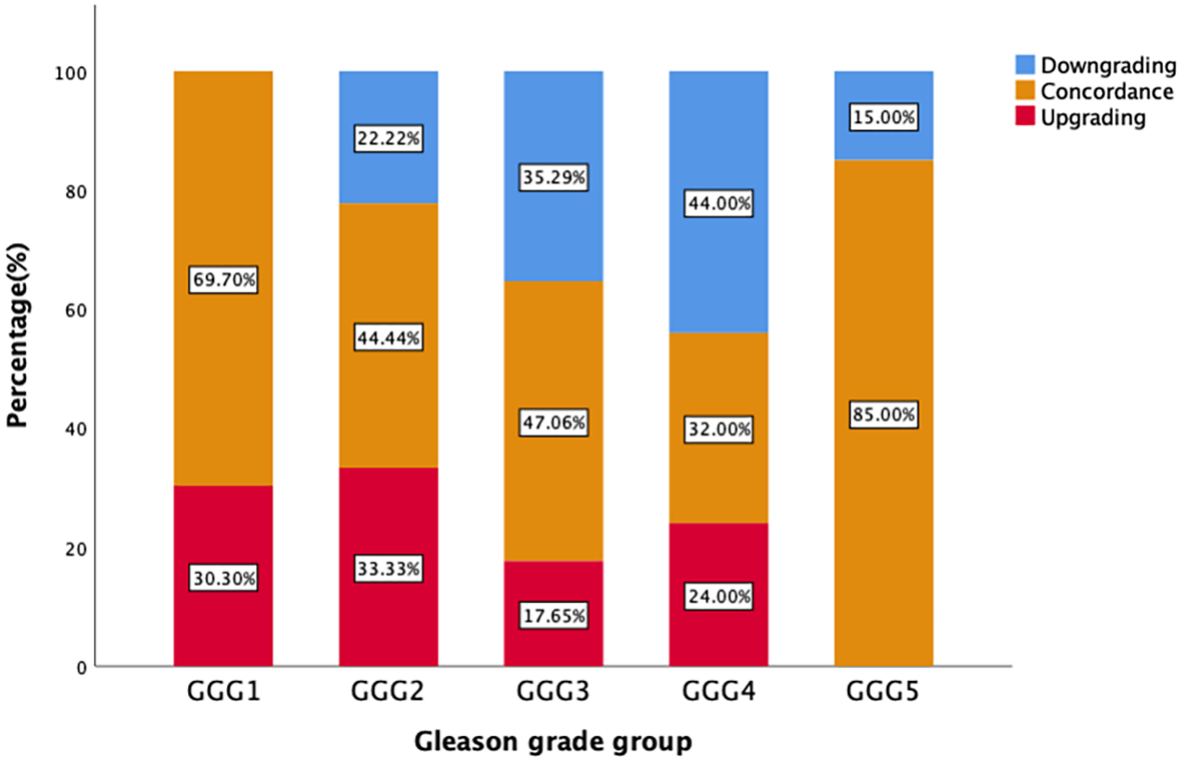
Distribution graph of change of biopsy GGG after RP.

**Figure 2** demonstrates that the upgrading rates of prostate biopsy groups GGG1, GGG2, GGG3, GGG4, and GGG5 after RP were 30.3% (10/33), 33.3% (6/18), 17.7% (3/17), 24.0% (6/25), and 0% (0/20), respectively. The degrading rates of prostate biopsy groups GGG1, GGG2, GGG3, GGG4, and GGG5 after RP were 0% (0/33), 22.2% (4/18), 35.3% (6/17), 44.0% (11/25), and 15.0% (3/20), respectively. Meanwhile, 69.7% (23/33) of biopsy results of GGG1, 44.4% (8/18) of GGG2, 47.1% (8/17) of GGG3, 32.0% (8/25) of GGG4, and 85.0% (17/20) of GGG5 remained concordant after RP.

### The predicting factors of GGGU and GGG degrading

Univariate logistic regression of parameters age, tPSA, PSAD, pGS, sGS, GPC, and number of positive cores was carried out separately for variable selection. Univariate logistic analysis showed that pGS [odds ratio (OR = 0.36, *p* = 0.01], GPC (OR = 1.03, *p <* 0.01), and number of positive cores (OR = 1.24, *p* = 0.04) were independent factors for predicting GGGU after RP, with sGS (OR = 0.58, *p* = 0.13) as a candidate variable (**Table 3**). Meanwhile pGS (OR = 2.09, *p* = 0.03) was the predicting factor for GGG degrading after RP. Firth’s bias-reduced multivariate logistic regression showed stable results (**Table 4**).

**Table 3:** Univariate logistic regression for predicting GGGU or GGG degrading after RP.

Variables	Candidate predicting variables	OR (95% CI)	*p*-value
GGGU	Age	1.04 (0.96, 1.14)	0.35
	tPSA	1.00 (0.98, 1.03)	0.72
	PSAD	0.97 (0.38, 2.18)	0.95
	pGS	0.36 (0.15, 0.78)	0.01
	sGS	0.58 (0.27, 1.15)	0.13
	GPC	1.03 (1.01, 1.06)	<0.01
	Number of positive cores	1.24 (1.02, 1.52)	0.04
GGG degrading	Age	1.04 (0.96, 1.14)	0.32
	tPSA	1.01 (0.98, 1.03)	0.65
	PSAD	1.24 (0.52, 2.74)	0.61
	pGS	2.09 (1.07, 4.22)	0.03
	sGS	1.40 (0.73, 2.67)	0.31
	GPC	0.99 (0.98, 1.01)	0.41
	Number of positive cores	1.05 (0.85, 1.28)	0.66

**Table 4:** Firth’s bias-reduced multivariate logistic regression for predicting GGGU after RP.

Variables	Candidate predicting variables	OR (95% CI)	*p*-value
GGGU	Age	1.05 (0.95, 1.16)	0.31
	PSAD	0.56 (0.17, 1.64)	0.30
	pGS	0.22 (0.07, 0.60)	<0.01
	sGS	0.49 (0.19, 1.19)	0.12
	GPC	1.04 (1.02, 1.07)	<0.01
	Number of positive cores	1.25 (0.94, 1.70)	0.13

### Nomogram establishment using variables of combined systematic and CEUS-targeted prostate biopsy

**Figure 3:**
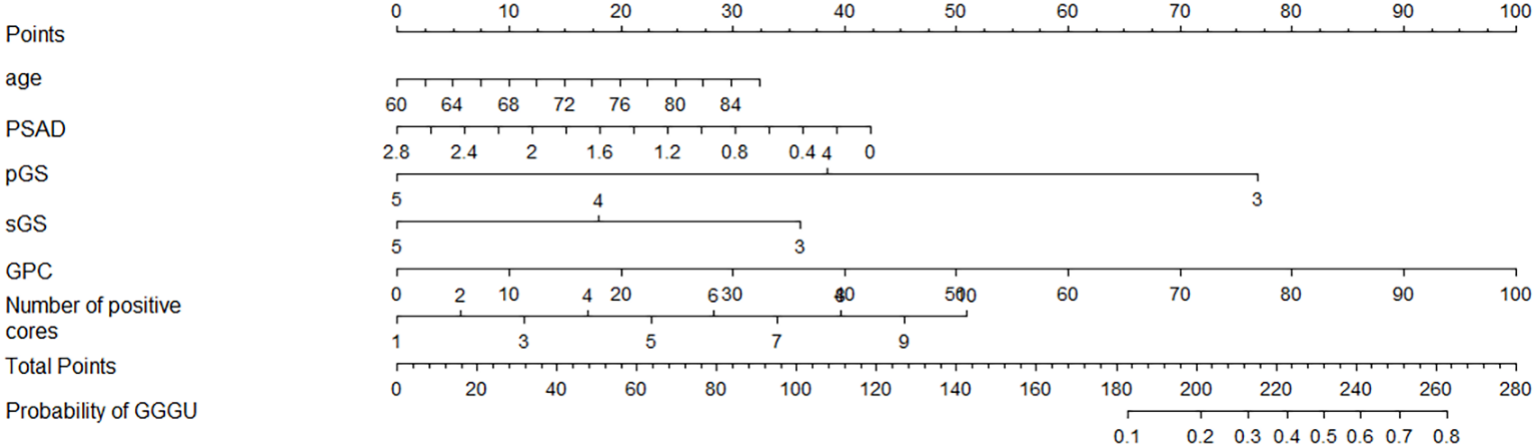
Nomogram for predicting GGGU after RP.

Four independent influencing factors were selected according to univariate logistic analysis, together with clinical indicators age and PSAD, which were closely related to the occurrence and risk stratification of prostate cancer based on previous studies (4–6); these six variables were used to construct a nomogram for predicting GGGU after RP (**Figure 3**).

For example, the preoperative data of an 80-year-old low-risk patient with prostate cancer were as follows: PSAD = 1.4, pGS = 3, sGS = 3, GPC = 60%, and number of positive cores = 6. By substituting these indicators into the nomogram, the predicted probability of GGGU occurring in this patient after RP can be obtained, as shown in **Supplementary Figure 1**: 25 points (age) + 21 points (PSAD) + 77 points (pGS) + 36 points (sGS) + 60 points (GPC) + 28 points (number of positive cores) = 247 points. The predicted probability of GGGU after RP for this patient is approximately 0.7, which indicates that the probability of pathological group escalation in this low-risk patient with prostate cancer is relatively high and, thus, is suitable for radical surgery rather than active monitoring or brachytherapy.

### The diagnostic performance of the nomogram

**Figure 4:**
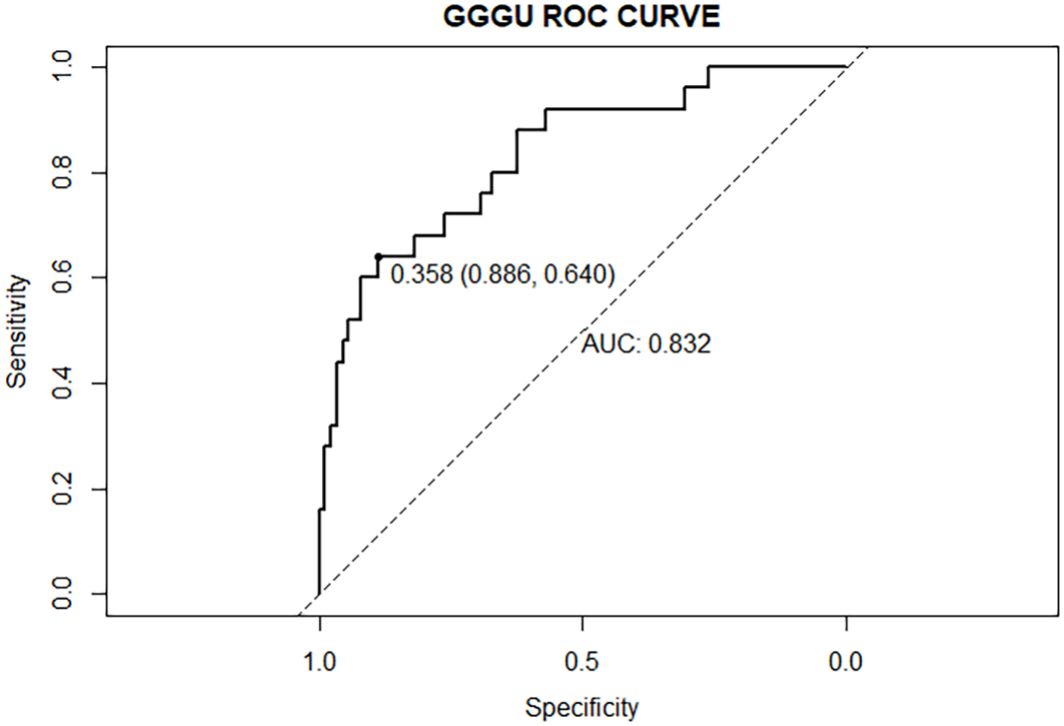
ROC curve of GGGU after RP predicted by the nomogram model.

**Figure 5:**
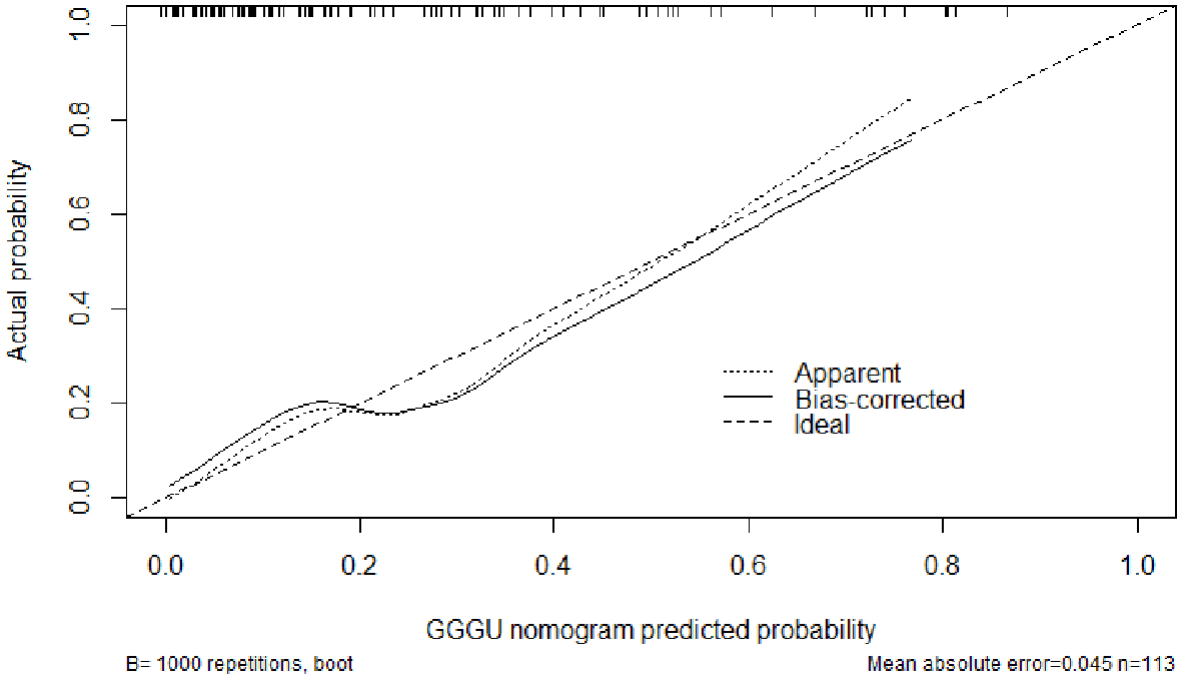
Calibration curve of the nomogram model for predicting GGGU post-RP.

The AUC of the nomogram predicting GGGU after RP was 0.83, and the calibration curve demonstrated good consistency of this nomogram prediction model, with a mean absolute error of 0.05 after 1,000 bootstraps (**Figures 4**, **5**). The results of leave-one-out cross-validation also demonstrated robustness: accuracy = 0.80 and mean squared error = 0.14.

## Discussion

The latest version of prostate cancer grouping and grading system was proposed at the conference of ISUP in November 2014 (12). Previous studies based on this new grouping system demonstrated that the 2014 ISUP version of the prostate cancer grading and grouping system was of great value in risk stratification, therapeutic decision-making, and prognostic accuracy (13). Consequently, it has been rapidly promoted and applied globally, and was accepted by the World Health Organization in 2016. Despite the fact that the 2014 ISUP grouping system can reduce the incidence of prostate cancer GGGU after RP, some patients still presented with clinically significant GGGU, which was defined as an increase from GGG ≤2 to ≥3 and from 3 to ≥4 (14). A nomogram based on the 2014 ISUP grouping system contributes to achieve the greatest possible consistency between the pathological GGG of prostate puncture biopsy and that of RP, which will assist clinicians in making accurate treatment decisions. Meanwhile, this study also paid attention to the GGG increase from 4 to 5.

The GGG obtained by MRI-guided targeted prostate biopsy is regarded as the gold standard for preoperative grading of prostate cancer (5). However, MRI-guided biopsy is expensive, time-consuming, and highly operator-dependent, which makes it difficult to apply in primary hospitals; instead, ultrasound-guided systematic biopsy is commonly used in clinical practice. The results of our 113 patients with prostate cancer showed that the pathological GGG of prostate puncture biopsy after RP was not completely consistent, with GGGU occurring in a total of 25 cases (22.1%) and GGG degrading in 24 cases (21.3%), while the remaining 64 cases (56. 6%) remained constant. Prostate cancer is characterized by abundant neovascularization and poor differentiation compared with normal prostate tissues (15). In our study, with the advantage of combined systematic and CEUS-targeted prostate biopsy, the pre-RP pathological GGG was in moderate agreement with post-RP GGG (Kappa = 0.46, *p <* 0.01), which was higher than that of other studies (16–18). CEUS can help to detect more suspicious prostate cancer foci (8), making the pre-RP GGG more accurate (19). Studies have shown that CEUS-guided targeted biopsy is similar to MRI-guided targeted biopsy in terms of preoperative GGG assessment (20).

Previous studies showed that potential predictors of GGGU after RP include age, body mass index (BMI), tPSA level, prostate volume, PSAD, number of positive puncture needles, GPC, pGS, sGS, preoperative imaging findings, and clinical stage (16). In this study, we found that GPC and pGS were independent factors of GGGU after RP. A lower pGS and a higher GPC indicated a higher probability of GGGU after RP, which was consistent with previous studies (17, 21). GPC was positively correlated with tumor volume and, thus, was commonly used to evaluate the size and range of prostate cancer (22). The larger prostate tumor volume might indicate a higher burden of prostate cancer, with a greater likelihood of high-grade tumor, which might be missed by puncture biopsy, thus leading to the occurrence of GGGU after RP. Although there was no significant correlation between sGS and GGGU after RP in this study (*p* = 0.13), which may be caused by sampling error, sGS was included in the subsequent nomogram analysis. The AUC of the nomogram with multiple predictors for predicting GGGU after RP was 0.83, and the calibration curve fitted well with the standard curve after internal validation, with a mean absolute error of 0.05 after 1,000 bootstraps, which indicated that the nomogram of this study had good consistency.

The limitations of this study were as follows: first, this study had a retrospective design; second, the sample size was not large; and third, some clinicopathological data were missing, causing potential bias.

In summary, good consistency of prostate cancer GGG was observed between combined systematic and CEUS-targeted prostate biopsy and specimens after RP. GPC and pGS were independent factors in predicting GGGU after RP, and the nomogram established on multiple risk factors for predicting GGGU after RP had good predictive efficacy.

## Data Availability

The raw data supporting the conclusions of this article will be made available by the authors, without undue reservation.

## Supplementary material

Supplementary Figure 1 Example of nomogram application for predicting GGGU after RP.
