# Supplementary figures and images for: The nomogram of contrast-enhanced ultrasound-targeted fusion biopsy predicts the pathology upgrade in prostate cancer
Source: Front Oncol. 2025 Nov 17;15:1632501. doi: 10.3389/fonc.2025.1632501 (PMC12665579; doi:10.3389/fonc.2025.1632501)

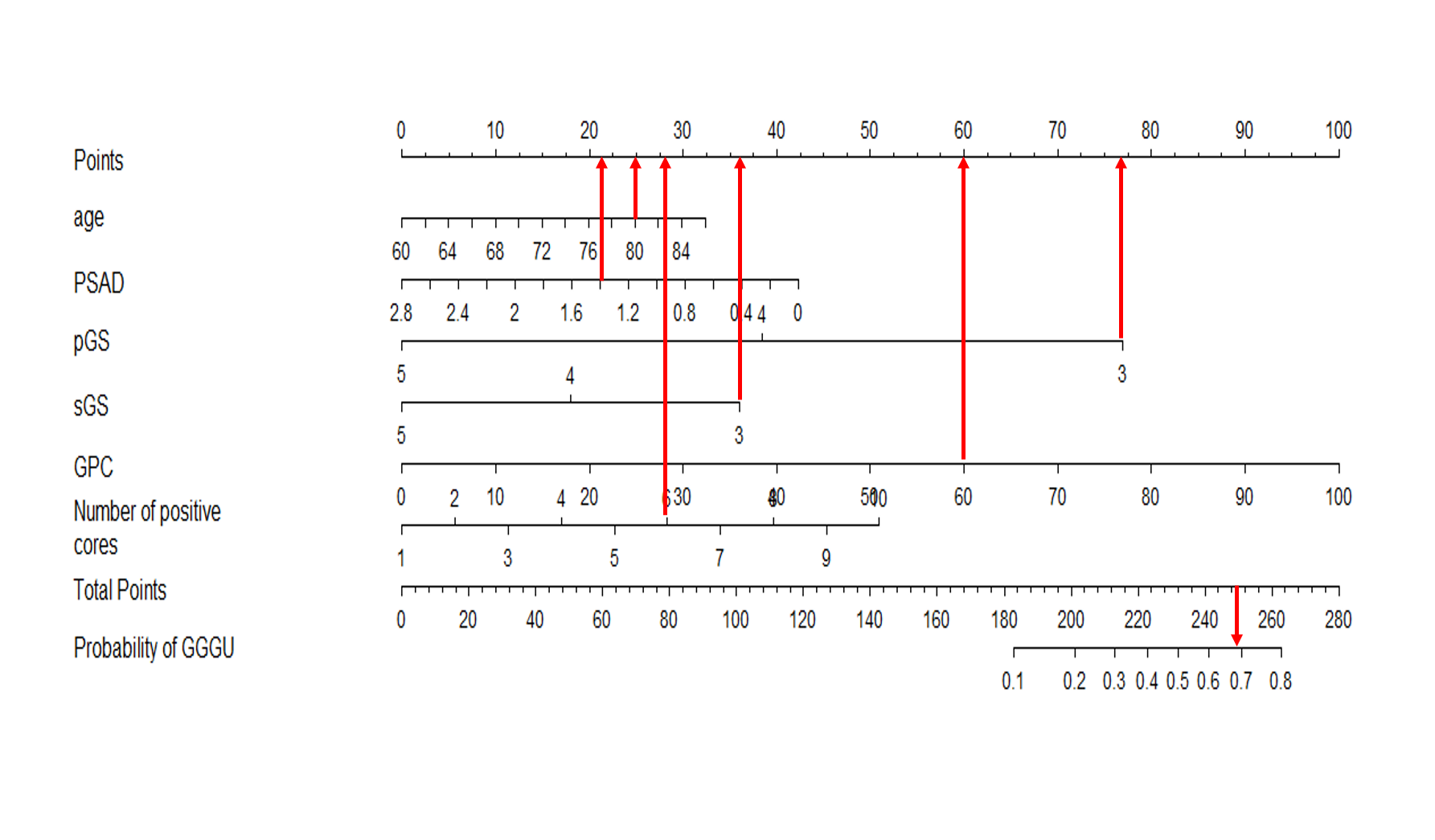

Supplement: Supplementary Figure 1 — Example of nomogram application for predicting GGGU after RP. [file Image1.tif]
